# FFR-CT is extremely useful in detecting hemodynamically significant left-main coronary artery stenosis with right coronary artery hypoplasia: A case report

**DOI:** 10.1016/j.jccase.2023.04.009

**Published:** 2023-06-01

**Authors:** Soji Kohyama, Yuichi Sawayama, Kohei Asada, Yousuke Higo, Kenji Kodama, Noriaki Yagi, Megumi Fukuyama, Atsushi Hayashi, Wataru Shioyama, Hiroshi Sakai, Tomoya Ozawa, Yoshihisa Nakagawa

**Affiliations:** Department of Cardiovascular Medicine, Shiga University of Medical Science, Shiga, Japan

**Keywords:** Fractional flow reserve from computed tomography, Left main coronary artery disease, Myocardial perfusion scintigraphy, Right coronary artery hypoplasia

## Abstract

Left main coronary artery (LMCA) stenosis in patients with coronary artery disease (CAD) is associated with a significant increase in cardiac events, and determining its contribution to ischemia is essential. Currently, several noninvasive modalities are available for the ischemic assessment of CAD. In multi-vessel disease, including LMCA disease, the accuracy of myocardial perfusion scintigraphy (MPS) for detecting myocardial ischemia can be poor. Fractional flow reserve from computed tomography (FFR-CT) has emerged as a promising noninvasive modality that can provide functional myocardial ischemia information. Herein, we describe the case of a 50-year-old woman with type 2 diabetes who presented to the hospital due to intermittent chest pain on exertion. Coronary computed tomography angiography showed right coronary artery hypoplasia, 25 % stenosis in the LMCA, and 75 % stenosis in the left anterior descending. FFR-CT identified myocardial ischemia due to LMCA stenosis, but MPS did not. Invasive coronary angiography with conventional fractional flow reserve was mostly consistent with the results of FFR-CT.

**Learning objective:**

Fractional flow reserve from computed tomography (FFR-CT), which is a novel noninvasive method, can provide absolute, not relative, functional myocardial ischemia information by applying computational fluid dynamics to coronary computed tomography angiography on a lesion-by-lesion basis. FFR-CT can be extremely useful in detecting patients with left main coronary artery stenosis with right coronary artery hypoplasia.

## Introduction

Left main coronary artery (LMCA) stenosis in patients with coronary artery disease (CAD) is associated with a significant increase in cardiac events [1], and determining its contribution to ischemia is essential. Myocardial perfusion scintigraphy (MPS) is widely used as a specific method for noninvasive imaging of regional myocardial ischemia [2]. However, the accuracy for detecting multi-vessel disease (MVD) or LMCA disease can be poor because it evaluates relative myocardial perfusion with its own other coronary arteries [3]. Fractional flow reserve from computed tomography (FFR-CT) has emerged as a promising noninvasive modality alternative to conventional fractional flow reserve (FFR). This enables the prediction of blood flow and pressure fields in coronary arteries and the calculation of lesion-specific FFR by applying computational fluid dynamics to coronary computed tomography angiography (CCTA) [4]. FFR-CT might be useful even in patients with MVD or LMCA disease because it provides absolute, not relative, functional myocardial ischemia detail based on anatomical information on a lesion-by-lesion basis. In this report, we present the case of LMCA stenosis with right coronary artery (RCA) hypoplasia in which myocardial ischemia could be identified by FFR-CT but not by MPS.

## Case report

**Fig. 1:**
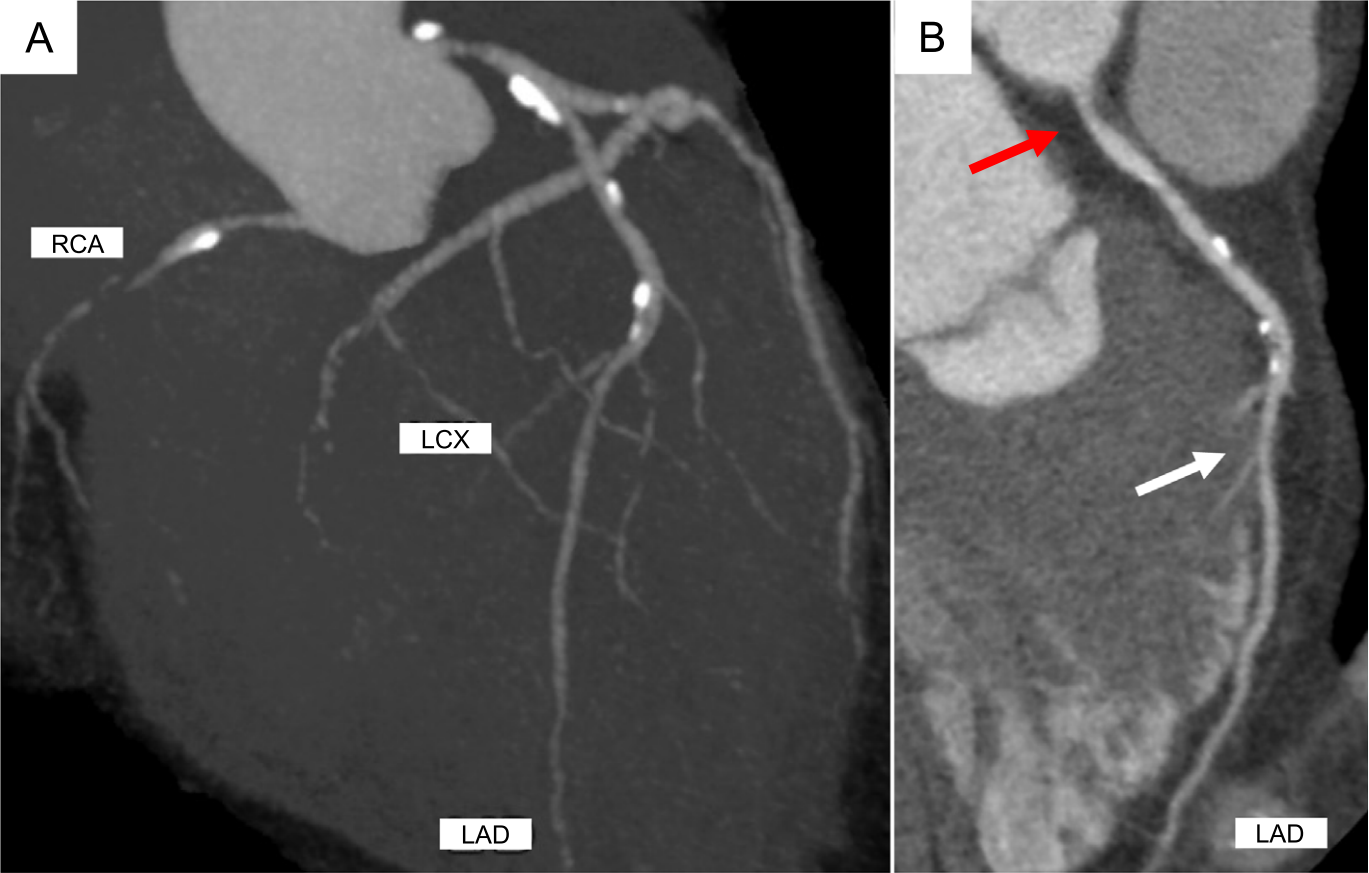
Findings in coronary computed tomography angiography. (A) Maximum intensity projection reformation of coronary computed tomography angiography shows right coronary artery hypoplasia. (B) Curved planer reconstruction of left anterior descending (LAD) shows 25 % stenosis in the left main coronary artery (LMCA), and 75 % stenosis in the LAD. The red arrows indicate the LMCA stenosis and the white arrows indicate the LAD stenosis. LCX, left coronary circumflex; RCA, right coronary artery.

**Fig. 2:**
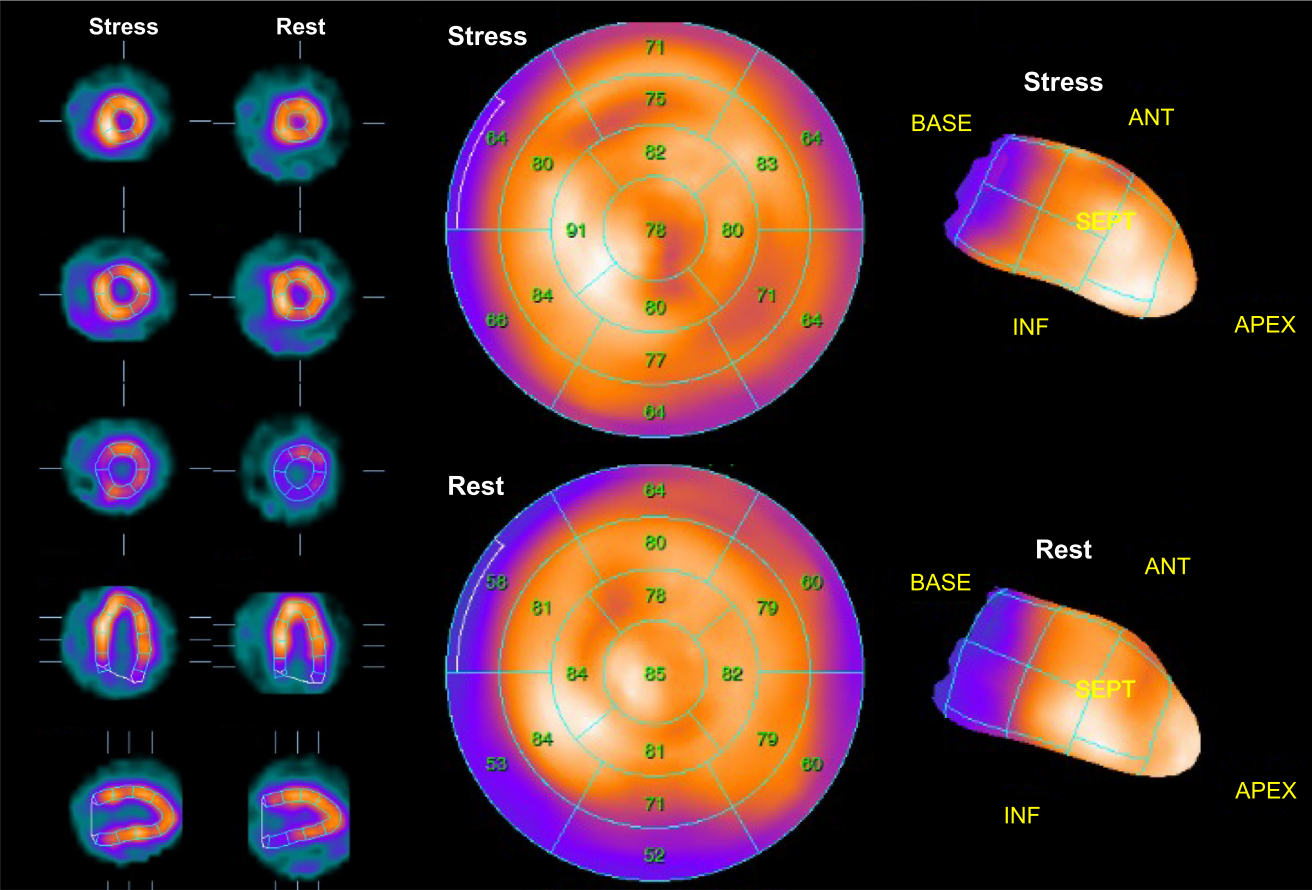
Adenosine stress thallium-201 myocardial perfusion scintigraphy. A normal perfusion pattern was observed (summed stress score = 3; summed rest score = 0; summed difference score = 3; stress defect = 4 %; transient ischemic dilatation ratio = 1.20).

**Fig. 3:**
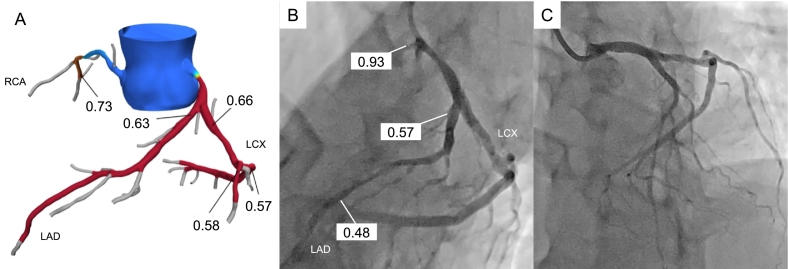
Coronary artery findings in FFR-CT, invasive FFR, and post percutaneous coronary intervention for the LMCA. (A) Fractional flow reserve from computed tomography (FFR-CT) showed hemodynamically significant stenosis in the left main coronary artery (LMCA). (B) The invasive fractional flow reserve (FFR) value decreased to 0.57 at distal site of the LMCA. (C) Invasive coronary angiography after deployment of a drug-eluting stent in the LMCA-LAD. LAD, left anterior descending; LCX, left coronary circumflex; RCA, right coronary artery.

A 50-year-old woman with type 2 diabetes mellitus presented to the hospital due to intermittent chest pain on exertion. Based on the results of blood tests (cardiac troponin was negative), a 12‑lead electrocardiogram (no remarkable ST change), and transthoracic echocardiography (no wall motion abnormalities), as well as the patient's typical chest pain and a coronary risk factor, she underwent CCTA in suspicion of CAD. CCTA showed RCA hypoplasia, 25 % stenosis in the LMCA, and 75 % stenosis in the left anterior descending (LAD; Fig. 1A,B). Therefore, we performed a further evaluation, which comprised adenosine stress thallium-201 MPS and FFR-CT. The MPS showed no major ischemic findings [summed difference score = 3, stress defect = 4 %, transient ischemic dilation (TID) ratio = 1.20; Fig. 2]. The FFR-CT analysis (cut-off value for ischemia: ≤0.80) showed that the decrease in value due to the LMCA stenosis was hemodynamically significant at 0.63 (Fig. 3A). Subsequently, invasive coronary angiography was performed, which showed 50 % stenosis at the LMCA and 75 % stenosis at the LAD. The FFR value decreased to 0.57 at the distal site of the LMCA lesion, which was largely consistent with the results of FFR-CT (Fig. 3B). In comparison, the FFR value decreased from 0.57 to 0.48 at the distal site of the LAD lesion, which was not hemodynamically significant (Fig. 3B). She underwent percutaneous coronary intervention for the LMCA (Fig. 3C). Deployment of one drug-eluting stent in the LMCA-LAD relieved her symptoms. The patient had an uneventful postoperative course during her hospital stay and had no evidence of recurrence after discharge.

## Discussion

The findings in this case have the following important clinical implications. In patients with LMCA stenosis and RCA hypoplasia, there were no major ischemic findings on MPS. In contrast, FFR-CT could identify any hemodynamically significant LMCA stenosis, even in such a background.

In stable CAD, revascularization therapy is only effective for coronary artery stenosis with proven ischemia. MPS is widely used for the noninvasive evaluation of regional myocardial ischemia, with a diagnostic accuracy of over 80 % [2]. Indeed, the current guidelines recommend noninvasive functional imaging, including MPS, for the diagnosis and risk assessment of stable CAD [5]. However, MPS is compromised in LMCA disease or MVD because it evaluates relative myocardial perfusion. Clinically, patients with LMCA disease with RCA stenosis have relatively normal perfusion on MPS in approximately half of them [3]. In our case, LMCA stenosis with RCA hypoplasia may have resulted in balanced ischemia, leading to the normal perfusion pattern on MPS. TID ratio (cut-off value for ischemia >1.23) on MPS is also used as a marker of the severity of CAD [6]. However, Lester et al. reported that the higher TID ratio did not reflect the myocardial ischemia in patients with normal perfusion patterns on MPS [6]. Therefore, the TID ratio in our case may not be a candidate for evaluating ischemia.

FFR is an index of the physiological significance of coronary artery stenosis and is defined as the ratio of maximal blood flow in a stenotic artery to normal maximal flow. It can be measured by invasive coronary angiography with >90 % accuracy in identifying ischemia-causing coronary artery stenosis [7]. Because angiography is a poor reflector of physiology, this approach is known to be superior to treatment strategies based on the degree of angiographic stenosis in reducing major adverse cardiac events [8]. LMCA stenosis in patients with CAD is associated with a considerable increase in cardiac events [1], and determining its contribution to ischemia is essential. However, the LMCA is one of the most challenging lesions to visually assess for hemodynamically significant stenosis. Especially in LMCA lesions, the discrepancy between the degree of angiographic stenosis and FFR values is often observed [9]. Hamilos et al. reported that the long-term clinical outcome of patients with an LMCA stenosis in whom revascularization therapy was deferred on the basis of FFR values >0.80 was acceptable [9]. Accordingly, for LMCA lesions, a treatment strategy based on FFR assessment may be important. FFR-CT has emerged as a noninvasive method alternative to the FFR, which is a novel method that enables prediction of blood flow and pressure fields in coronary arteries and calculation of lesion-specific FFR by applying computational fluid dynamics to CCTA [4]. FFR-CT has higher accuracy of diagnostic performance for detecting ischemia-causing stenosis than CCTA and a good correlation with invasive FFR values [4]. Moreover, in patients with MVD, FFR-CT has a higher sensitivity (FFR-CT: 93 % vs. MPS: 51 %) and a lower false negative value (FFR-CT: 7 % vs. MPS: 49 %) than MPS for detecting myocardial ischemia [10]. In this context, FFR-CT may be more useful for MVD or LMCA lesions than MPS to identify ischemia-causing stenosis. However, to date, there have been no reports regarding LMCA stenosis with RCA hypoplasia showing typical balanced ischemia, which could be detected by FFR-CT, but could not by MPS.

In conclusion, we present a case in which FFR-CT, but not MPS, could identify myocardial ischemia due to LMCA stenosis in a patient with RCA hypoplasia. FFR-CT can be extremely useful in detecting patients with extensive LMCA disease.

## Consent statement

Informed consent was obtained from the patient for publication of this case report.

## Declaration of competing interest

The authors declare no conflict of interest associated with this manuscript.
